# Latitudinal drivers of oyster mortality: deciphering host, pathogen and environmental risk factors

**DOI:** 10.1038/s41598-020-64086-1

**Published:** 2020-04-29

**Authors:** Elodie Fleury, Pierrick Barbier, Bruno Petton, Julien Normand, Yoann Thomas, Stéphane Pouvreau, Gaétan Daigle, Fabrice Pernet

**Affiliations:** 1 Univ Brest, Ifremer, CNRS, IRD, LEMAR, F-29280 Plouzane, France; 2 Ifremer, Laboratoire Environnement Ressources de Normandie, 14520 Port en Bessin, France; 3 Département de Mathématiques et Statistique, Université Laval, Sainte-Foy, Québec, G1K 7P4 Canada

**Keywords:** Ecosystem ecology, Ecological epidemiology

## Abstract

Diseases pose an ongoing threat to aquaculture, fisheries and conservation of marine species, and determination of risk factors of disease is crucial for management. Our objective was to decipher the effects of host, pathogen and environmental factors on disease-induced mortality of Pacific oysters (*Crassostrea gigas*) across a latitudinal gradient. We deployed young and adult oysters at 13 sites in France and we monitored survival, pathogens and environmental parameters. The young oysters came from either the wild collection or the hatchery while the adults were from the wild only. We then used Cox regression models to investigate the effect of latitude, site, environmental factors and origin on mortality risk and to extrapolate this mortality risk to the distribution limits of the species in Europe. We found that seawater temperature, food level, sea level atmospheric pressure, rainfall and wind speed were associated with mortality risk. Their effect on hatchery oysters was generally higher than on wild animals, probably reflecting that hatchery oysters were free of Ostreid herpesvirus 1 (OsHV-1) whereas those from the wild were asymptomatic carriers. The risk factors involved in young and adult oyster mortalities were different, reflecting distinct diseases. Mortality risk increases from 0 to 90% with decreasing latitude for young hatchery oysters, but not for young wild oysters or adults. Mortality risk was higher in wild oysters than in hatchery ones at latitude > 47.6°N while this was the opposite at lower latitude. Therefore, latitudinal gradient alters disease-induced mortality risk but interacts with the initial health status of the host and the pathogen involved. Practically, we suggest that mortality can be mitigated by using hatchery oysters in north and wild collected oysters in the south.

## Introduction

Diseases pose an ongoing threat to aquaculture, fisheries and conservation of marine species^1,2^. One of the most striking examples is illustrated by massive mortality events occurring in the Pacific oyster (*Crassostrea gigas*), one of the major invertebrate species harvested globally^3–5^. Since 2008, massive mortality events in *C. gigas* have been reported in almost all farming areas in France^6^ and were associated with the detection of a new genotype of Ostreid herpesvirus 1 called μVar^7^. Between 2008 and 2014, the geographical distribution of OsHV-1 μVar-related mortalities of oysters has expanded along the European coastline from Portugal to Norway, and closely related variants of OsHV-1 have been detected in Australia, New Zealand and Asia^3–5^.

In Europe, OsHV-1 outbreaks every year when seawater temperature is between 16 °C and 24 °C^8,9^. Infection starts when viral particles come into contact with susceptible hosts. There is a threshold dose for infection and a dose-response effect of OsHV-1 on mortality^10,11^. The infected host shed viral particles into the water column and dispersal to new hosts occurs via water currents^8,12,13^. Mortality starts in the intertidal farming areas and spread off-coast in a way that depends of food quantity and quality, turbidity, growth rate and energetic reserves of oysters^14–16^. Latent and asymptomatic OsHV-1 infections are able to persist in hosts^17^. In this case, virus reactivation can occur several weeks to months after initial exposure.

The susceptibility of oysters decreases with age for OsHV-1^13,18,19^. It increases, however, for *Vibrio aestuarianus*^19^, a pathogenic bacterium that cause major mortality in adult oysters^3,4^. The risk factors involved in young and adult oyster mortalities are partly different, reflecting differences in the interactions with these distinct pathogens^14^. For adult oysters, mortality is primarily associated with connectivity to channel rivers and salinity, and secondarily with connectivity to oyster farms^14^.

Most of the studies aimed at defining mortality risk factors for oysters were conducted at the local (<10 km^2^) or regional scale (10–1000 km^2^), but not at broader scales. Measurements made in different scales are often not comparable, and correlations among variables that are evident at a given scale may disappear or change sign when the scale is extended^20^. As most host-pathogen interactions are highly vulnerable to changes in environment^1^, latitudinal gradient and habitat characteristics alter the likelihood of disease outbreaks by influencing the host, the pathogen, or both^21,22^. It is therefore plausible, or even likely, that expanding the scale of observation of oyster mortality would provide new information on disease risk factors.

The overall objective of our study was to decipher the interactive effects of host, pathogen and environmental factors on disease-induced mortality of oysters across a latitudinal gradient. More specifically, we developed mortality risk models based on latitude, environmental factors and pathogen detection in young (<1 year old) and adult oysters (>18 months). The young oysters came from either the wild collection or the hatchery while the adults came only from the wild only. For instance, young oysters from the wild are often asymptomatic carriers of the virus whereas hatchery oysters are generally free of OsHV-1^13^, and the initial health status of oysters interacts with environment on mortality risk^23^. Oysters were deployed at 13 contrasted sites, covering a broad range of 600 km and 7° of latitude, including the English Channel, the Bay of Biscay (French Atlantic coast) and a Mediterranean lagoon. Although the biogeographic distribution of *C. gigas* (and its pathogens) is much larger than that covered by our study, particularly because of its northward expansion in recent decades^24^, we have included a large gradient of temperature and sites where oysters may or may not complete their reproductive cycle. Survival was monitored during one year along with pathogen detection analyses (OsHV-1 and *V. aestuarianus*) and measurements of environmental parameters. We have chosen parameters that are known to influence the risk of disease at finer scales (seawater temperature, salinity, and food level) and others that have never been studied because they rely on a large-scale study framework (sea level atmospheric pressure, rainfall, wind speed and direction).

## Method

### OYSTERS

Oysters were part of the Ifremer observatory network (https://wwz.ifremer.fr/observatoire_conchylicole). Young oysters consisted of wild spat collected on limed tiles in Arcachon Bay during summer 2012 or 3-month-old animals produced by a private hatchery^23^. These two batches of oysters were gathered at the Ifremer laboratory (La Trinité-sur-Mer, France) before deployment in the field. A subsample of 50 oysters from each batch was screened for OsHV-1 DNA by qPCR and 300 individuals were exposed to a thermal elevation at 21 °C for 1 month in cohabitation with healthy spat to reveal both disease expression and transmission^13^. Wild oysters were asymptomatic carriers of OsHV-1 as the virus was detected in 16 out of 50 individuals and mortalities associated with OsHV-1 occurred in both the tested and the cohabited healthy oysters after thermal elevation. In contrast, oysters from the hatchery were considered specific pathogen-free because OsHV-1 DNA was not detected and no mortality occurred after thermal elevation.

Adult oysters were obtained from spat that settled on collectors during summer of 2011 near the Aix Island (Charente Maritime, France). Then, they were detached during spring 2012 and transferred in the Bay of Morlaix (Northern Brittany, France) where they suffered *ca*. 75% mortality presumably due to OsHV-1. Viral DNA was detected in 14 out of 50 samples. *V. aestuarianus* DNA was not detected in the three batches of oysters.

### Experimental Design and Sampling

**Figure 1:**
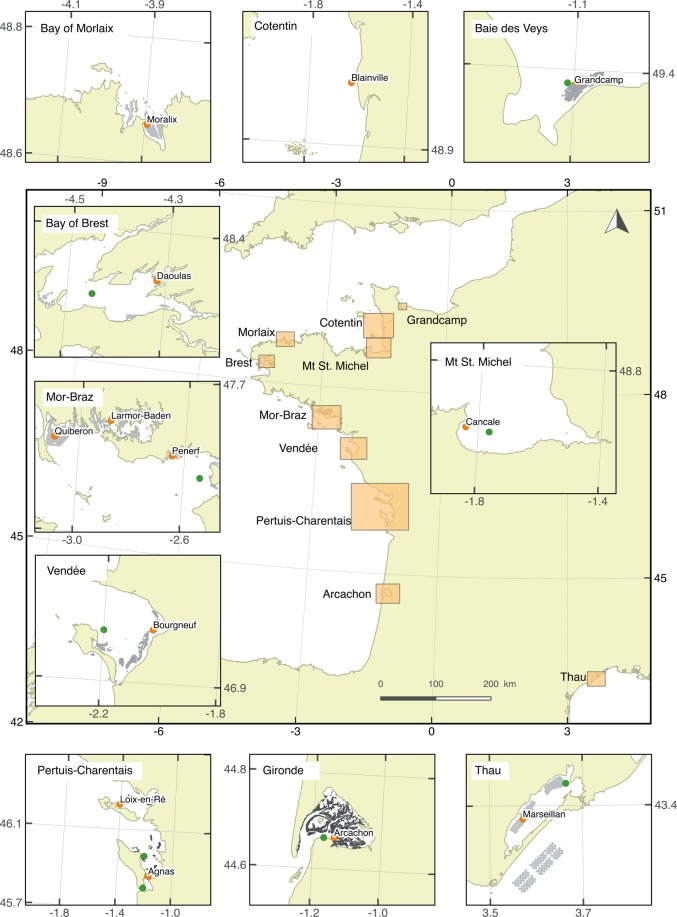
Site map. Orange circle: oyster, temperature and salinity measurements. Green circle: phytoplankton cell count. This map was done with the open source QGIS ver. 2.18.9. (QGIS Development Team, 2016, https://qgis.org).

The oysters were deployed at 13 sites on 11 March 2013, at least 1 month before the start of the annual OsHV-1 outbreak while seawater temperature was between 5.7 and 11.4 °C, and their survival was followed for 280 days until 16 December 2013. The selected sites were interspersed over a 600 km long latitudinal gradient, covering the English Channel, the Bay of Biscay (French Atlantic coast) and the Thau lagoon in the Mediterranean, between 43.3° and 49.3° latitude (Fig. 1, Table S1). Eleven sites were intertidal, situated 0.75 m above sea level, corresponding to 80% immersion time, and located at the center of the farming areas. The two other sites located in the Mediterranean Thau lagoon (Marseillan) and the Bay of Quiberon were subtidal, reflecting local farming practices. The effect of bathymetry (subtidal *vs*. intertidal) is confounded with the environmental factors and latitude.

Young oysters were held in one 45 × 45 × 8 cm mesh bag for each origin (6 mm mesh, 350 individuals per bag) while adults were in triplicate 90 × 45 × 8 cm mesh bags (10 mm mess, 300 oysters per bag). Oyster bags were attached to iron racks according to current practices. In the Bay of Quiberon, oysters were placed in sea cages, and in the Thau lagoon, young and adult oysters were held in pearlnets and Australian baskets respectively^8^. Each site was visited at least 17 times between deployment and the end of the monitoring (11 and 28 March, 10 and 26 April, 10 and 28 May, 10 and 27 June, 11 and 23 July, 8 and 22 August, 6 and 18 September, 8 October, 5 November, and 16 December) to measure oyster survival and collect samples for pathogen detection analyses. Dead and live individuals were counted, and the dead were removed from the bag. The samples for pathogen detection analyses were collected 10 times between 10 May and 18 September. Each sample consisted in three pools of three individuals. Detection and quantification of pathogen DNA were carried out by the Pole d’analyses et de recherche de Normandie (LABEO, Saint-Lô, France) using standard real-time PCR protocols^25,26^.

### Environmental data

For each site, temperature and salinity were directly measured at the vicinity of the oyster bags using a multiparameter probe (MP3, NKE instrumentation, Hennebont, France) (Fig. 1). Data were acquired every 30 minutes during the entire period of study. Daily means were calculated for each parameter. Phytoplanktonic cell concentrations, a proxy of the food concentration in the seawater, were obtained from the French Observation and Monitoring program for Phytoplankton and Hydrology in coastal water (REPHY). Phytoplankton cells were counted from seawater samples preserved with Lugol’s solution using an inverted microscope.

Meteorological data like 10-meter wind direction and speed, mean sea level atmospheric pressure, and rainfall were provided daily for each of the 13 locations from the ERA-Interim archive (http://apps.ecmwf.int/datasets/data/interim-full-daily/levtype=sfc/). ERA-Interim is a global atmospheric reanalysis which gives a numerical description of the recent climate, produced by combining models with observations. The ERA-Interim atmospheric model is configured with a reduced Gaussian grid with uniform 79-km spacing for grid-point fields. Data from the near neighbor grid-point for each of the studied locations were selected for analysis.

Wind data, which consisted of speed (Ws, m s^−1^) and direction (Wd, degree where 0° = North and 90° = West), were transformed into speed in the north-south (W_NS_) and west-east (W_WE_) axes according to the following formulas:

$${W}_{NS}=Ws\times \,\cos \left(Wd\times \frac{\arccos (-1)}{180}\right)$$

$${W}_{WE}=Ws\times \,\sin \left(Wd\times \frac{\arccos (-1)}{180}\right)$$

A high positive value of W_NS_ means that the wind comes from the north, whereas a high but negative value means that the wind is coming from the south. Similarly, a positive high value of W_WE_ means that the wind is coming from the west, whereas a high but negative value means that the wind is coming from the east.

### Statistical analyses

Non-parametric estimates of the survivor function were computed by the Kaplan–Meier method^27^. Survival time was measured as days from 11 March 2013, when oysters were deployed in each site. Combinations of batch × site were used as strata, and survival estimates among strata were compared by using the log-rank test.

The survival time curves of oysters of each batch were compared using Cox regression models^28^. In our study, the number of dead oysters was reported by time intervals so that the exact date of death of each individual was unknown (interval censored data). For each mortality observation, a death day was randomly assigned within the time interval. Each oyster bag was considered as a cluster to obtain robust parameter estimates. The proportionality of hazards was checked with Martingale residuals^29^. Because this assumption was violated, the analyses were stratified by age-classes (young versus adult).

For the young oysters, three types of Cox regression model were run. The first model tested the effect of latitude (or site), origin (wild vs. hatchery), and their interaction (model 1). We then used the model parameter estimates to predict survival probability and hazard ratio (wild vs. hatchery) at the northern and southern limits of the study area and extrapolated to the distribution limits of the species in Europe that is Gibraltar in the south and Norway in the north^24^. The other two models tested the effect of environmental variables taken at each site instead of site *per se*. Indeed, environment and site were confounded factors. We first evaluated the effect of environmental conditions that prevailed before the onset of mortalities on future mortality risk (model 2). To do this, we used the average of each environmental variable calculated over the longest period preceding the mortalities (15 d for wild and 50 d for hatchery oysters). Then, we evaluated the effect of the environmental conditions and pathogen load that prevailed during the mortality episode using a left-truncated right-censored Cox regression model with time-dependent covariates (model 3). We used average values of each environmental parameters calculated over each time interval. For models 2 and 3, environmental factors were first tested one by one (univariate analysis), and then significant explanatory covariates were selected using a stepwise method and tested in the multivariate regression model.

For adults, we conducted models 1 and 3 only. It was not possible to run model 2 because mortality started right after deployment. Missing environmental and pathogen data were handle by multiple imputation using fully conditional specification^30^. The data analysis was generated using SAS software, Version 9.4 of the SAS System for Window (SAS Institute Inc., Cary, NC, USA).

## Results

### Survival of oysters

**Figure 2:**
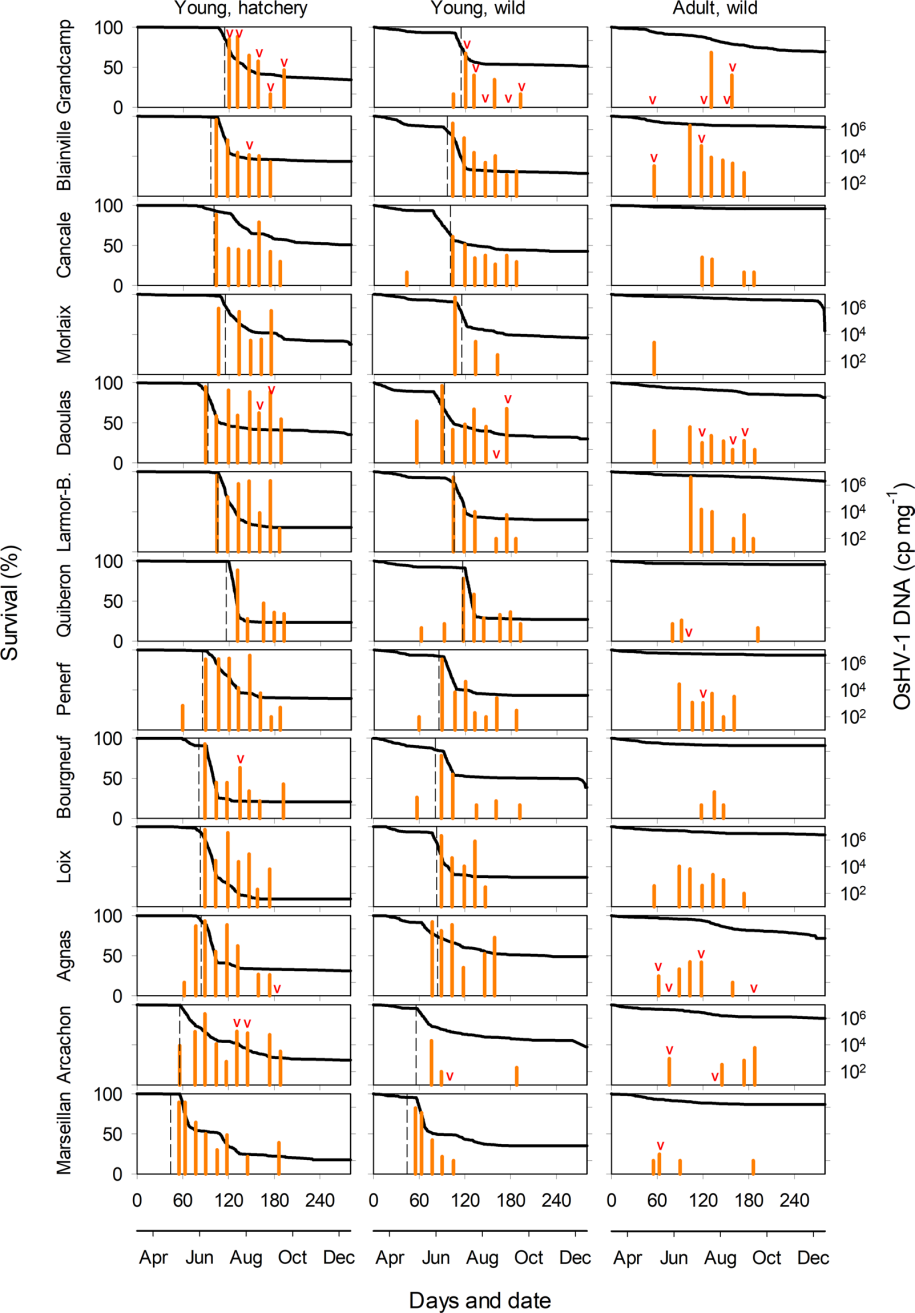
Survival curves of oysters (line) and viral load (bars) for each batch of oysters and sites. Viral load was expressed as mean level of OsHV-1 DNA copy mg^−1^ (n = 3 pools of 3 oysters). Dashed line on X axes indicate when seawater reached 16 °C. V indicates positive detection of *Vibrio aestuarianus* in oyster tissues.

Oysters were affected by mortalities at all sites. In young oysters, mortality generally occurred abruptly in late spring in the southern sites or early summer at the northernmost sites when seawater temperature reached ∼16 °C, whereas the phenomenon was more gradual in adults (Fig. 2). Young oysters showed lower survival than adults irrespective of site (log-rank test, p < 0.001). For instance, the final survival of young oysters varied from 9.8% to 51.2% (mean value of 35.4 ± 10.4% SD among sites) compared to 54.6 to 95.9% for adults (mean value of 83.6 ± 11.9%).

### Pathogen detection

OsHV-1 DNA was detected at all sites, mainly in young oysters (Fig. 2). For instance, 277/528 (52.5%) samples were positive for OsHV-1 in young oysters compared to only 70/264 in adults (26.5%). Detection usually occurred within the mortality period, except for wild oysters at five sites that were positive before this period. Levels of OsHV-1 DNA could reach >10^6^ DNA copies mg^−1^ during mortality events. Such a high level of detection was rarely achieved in adults.

Detection of *Vibrio aestuarianus* DNA was sporadic in space and time. Both young (47/711, 5.8%) and adult (27/371, 7.3%) *C. gigas* were occasionally positive. The highest detection of *V. aestuarianus* was observed at the northernmost site where 21/60 (35.0%) young oysters and 5/30 (16.7%) adults were positive.

### Environmental condition

**Figure 3:**
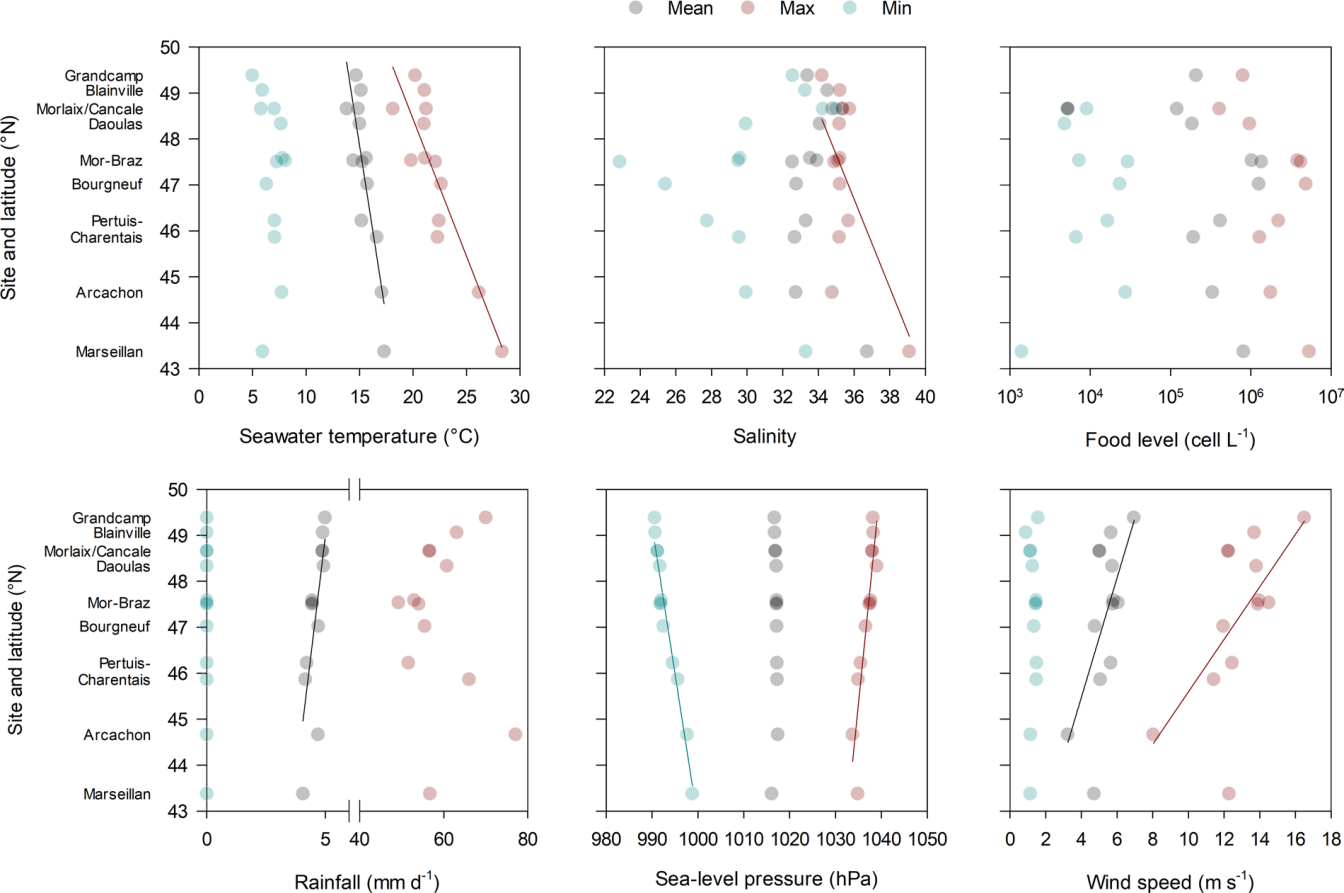
Environmental parameters used in Cox regression models in relation with latitude or sites.

The whole dataset of environmental parameters is available (repository data file and Figure S1). Average data are briefly described here as a function of latitude (Fig. 3). Parameters related to seawater temperature, salinity, rainfall, sea level atmospheric pressure, and wind speed varied with latitude. Mean and maximum seawater temperature increased southward respectively from 13.8 to 17.3 °C and from 18.1 to 28.3 °C (Fig. 3). The date at which seawater temperature reached 16 °C increased northward (days~10.8 × latitude − 418.1, r^2^ = 0.771, p < 0.001). The maximum recorded salinity increased southward, mainly due to the high values observed in the Thau lagoon (Marseillan), and was negatively correlated with mean rainfall (r^2^ = 0.302, p = 0.051). Mean sea level atmospheric pressure minima and maxima were negatively and positively correlated, respectively, with latitude. Rainfall was negatively correlated with sea level atmospheric pressure (r^2^ = 0.419, p < 0.001). The other parameters showed no latitudinal gradient despite sometimes large differences among sites. For example, salinity minima varied considerably from 22.8 to 35.0 with no relationship to latitude.

## Risk Analyses

### Young oysters

**Figure 4:**
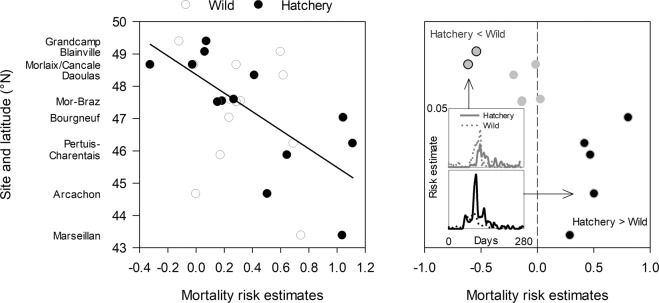
Mortality risk estimates of wild and hatchery oysters (left) and their differences (right) as a function of latitude or sites. Left panel: mortality risk increased southward for hatchery oysters (black line) but not for wild ones. Right panel: mortality risk was higher for wild than for hatchery oysters in two northern sites (grey circles with black edges), while the opposite was observed in the five southernmost sites (black circles with grey edges). The mortality risks were similar between origins in the middle of the study area (grey circles with no edge). Insets show mortality risk estimates as a function of time for the two northern sites (upper graph) and the five southernmost sites (lower graph).

**Figure 5:**
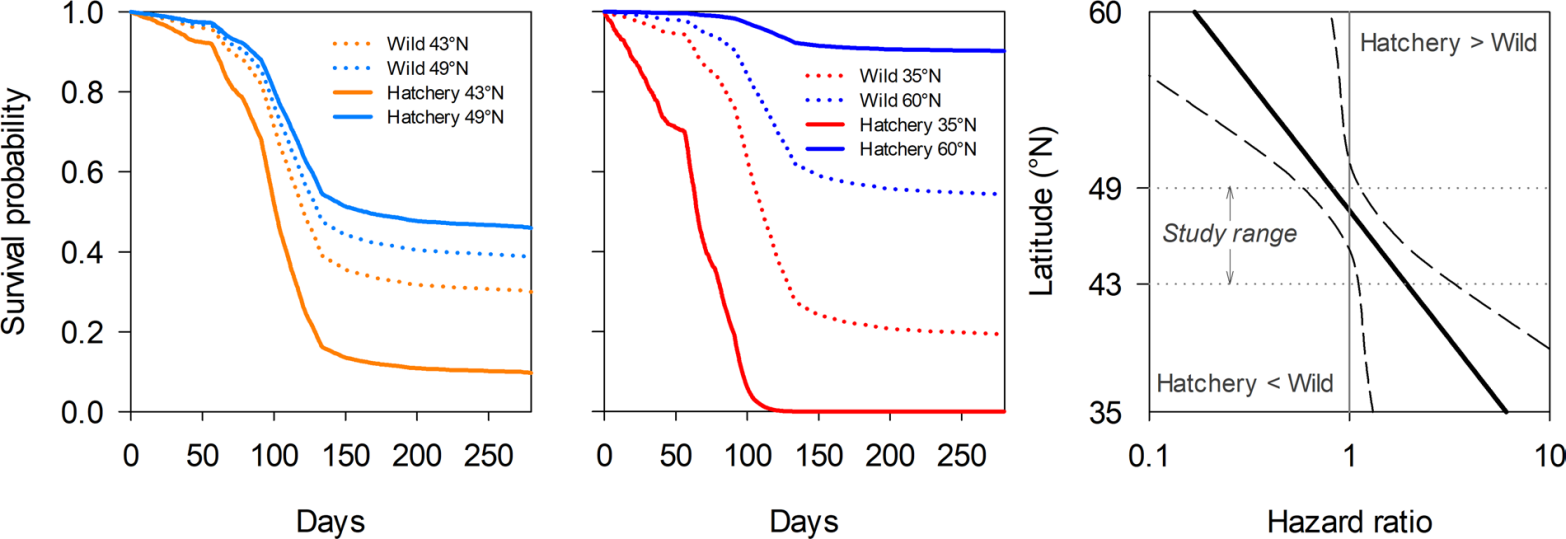
Predicted survival probability curves of wild and hatchery oysters as a function of latitude at the northern and southern limits of the study area (left) and extrapolated to the distribution limits of the species in Europe, Gibraltar and Norway (center). Hazard ratio of hatchery vs wild oysters as a function of latitude (right). Values predicted at latitude <43°N and >49°N are extrapolations.

Origin of young oysters interacted with latitude or site on mortality risk (Tables 1 and S1). While mortality risk of young wild oysters showed no latitudinal pattern, it was higher south of Brittany than in the north for hatchery oysters (Fig. 4). Any increase of 1° latitude reduced mortality risk by 17% in hatchery oysters compared to only 4% (a difference that was not significant) in wild oysters (Table 1). More particularly, mortality risk was higher in wild than in hatchery oysters in two northern sites, while the opposite was observed in the five southernmost sites (Fig. 4). In the two northern sites, mortality occurred *ca*. 14 d sooner in wild oysters than in hatchery ones, but not in the southernmost sites (see insets in Fig. 4). In the middle of the study area, the mortality risks were similar between oyster origins. Survival probabilities predicted by the model extrapolated to the distribution limits of the species in Europe varied from 0 to 90% for hatchery oysters and 19 to 54% for wild oysters (Fig. 5). The mortality risk was the same for both wild and hatchery oysters when latitude was 47.6°N (hazard ratio = 1.000, 95%CI [0.813–1.230], Fig. 5).

**Table 1:** Model parameter estimates from multivariate Cox regression models using latitude or environmental parameters selected by the stepwise procedure as covariates for young oysters. The average of each environmental variable was calculated over the longest period preceding the mortalities (15 d for wild and 50 d for hatchery oysters).

Parameter	Level	Df	Estimate	SE	χ^2^	p	Odds ratio	95% CI	
**Model 1: Latitude and origin**									
Latitude		1	−0.040	0.051	0.6	0.439			
Origin	Hatchery	1	6.823	2.971	5.3	0.022			
	Wild	0	0.000	.	.	.			
Latitude × origin	Hatchery	1	−0.143	0.063	5.2	0.022	0.83	0.78	0.89
	Wild	0	0.000	.	.	.	0.96	0.87	1.06
**Model 2: Environment over the period preceding the mortalities and origin**									
Origin	Hatchery	1	0.059	0.029	4.2	0.040			
	Wild	0	0.000	.	.	.			
Temperature (T)		1	−0.035	0.034	1.1	0.305			
Food level (F)		1	0.074	0.156	0.2	0.637			
Rainfall (R)		1	0.350	0.051	46.3	<0.001	1.42	1.28	1.57
Sea level pressure (SLP)		1	0.795	0.290	7.5	0.006			
Wind speed (W_NS_)		1	0.582	0.149	15.3	<0.001			
Origin × T	Hatchery	1	0.357	0.045	63.2	<0.001	1.38	1.30	1.46
	Wild	0	0.000	.	.	.	0.97	0.90	1.03
Origin × F	Hatchery	1	0.689	0.194	12.6	<0.001	2.15	1.69	2.73
	Wild	0	0.000	.	.	.	1.08	0.79	1.46
Origin × SLP	Hatchery	1	1.649	0.293	31.7	<0.001	11.52	6.80	19.52
	Wild	0	0.000	.	.	.	2.22	1.26	3.91
Origin × W_NS_	Hatchery	1	0.964	0.183	27.8	<0.001	4.70	3.62	6.09
	Wild	0	0.000	.	.	.	1.79	1.34	2.40

All environmental variables measured during the interval preceding mortality were associated with young oyster mortality risk, and most of them interacted with origin (Table S2). Seawater temperature, food quantity, sea level atmospheric pressure and wind speed (northerly oriented) were retained in the Cox multivariate regression model, and their effect on hatchery oyster mortality risk was consistently higher than that of wild animals (Table 1). Any increase in parameter value increased the risk of oyster mortality, but more strongly for hatchery versus wild animals. Moreover, temperature and food had no effect on the risk of mortality of wild oysters as opposed to hatchery ones. Precipitation was the only parameter that increased mortality risk in a similar way for both oyster origins.

All environmental variables measured during the mortality period were associated with young oyster mortality risk, and many of them interacted with origin (Table S3). OsHV-1 DNA, food level, and wind speed (northerly oriented) were retained in the time-dependent Cox regression model and their effect on oysters was the same irrespective of origin (Table 2). Any increase in parameter values increased the risk of oyster mortality. Also, temperature, salinity, sea level atmospheric pressure, and precipitation were retained by the model, but their effect on mortality risk varied with origin. Again, any increase in parameter values increased the risk of oyster mortality, but more strongly for hatchery than for wild animals. For example, a 1 °C increase in seawater temperature was associated with a 21% increase in mortality risk for hatchery oysters compared to a 7% increase for wild oysters. Overall, OsHV-1 was by far the most important factor associated with mortality risk in young oysters (highest χ^2^ value).

**Table 2:** Model parameter estimates from multivariate Cox regression models using time-dependent environmental parameters and pathogen detection selected by the stepwise procedure for young oysters.

Parameter	Level	Df	Estimate	SE	χ^2^	p	Odds ratio	95% CI	
Origin	Hatchery	1	−59.865	9.809	37.2	<0.001			
	Wild	0	0.000	.	.	.			
OsHV-1		1	0.001	0.000	1257.8	<0.001	1.00	1.00	1.00
*V. aestuarianus* (*Va*)		1	0.128	0.198	0.4	0.516			
Temperature (T)		1	0.069	0.011	39.7	<0.001			
Salinity (S)		1	−0.019	0.018	1.1	0.301			
Food level (F)		1	0.354	0.013	730.9	<0.001	1.43	1.39	1.46
Rainfall (R)		1	0.156	0.017	88.3	<0.001			
Sea level Pressure (SLP)		1	0.067	0.012	33.2	<0.001			
Wind speed (W_NS_)		1	0.107	0.020	29.9	<0.001	1.11	1.07	1.16
Origin × *Va*	Hatchery	1	0.536	0.228	5.5	0.019	1.94	1.51	2.51
	Wild	0	0.000	.	.	.	1.14	0.77	1.68
Origin × T	Hatchery	1	0.121	0.010	143.6	<0.001	1.21	1.19	1.23
	Wild	0	0.000	.	.	.	1.07	1.05	1.10
Origin × S	Hatchery	1	0.143	0.024	36.9	<0.001	1.13	1.10	1.17
	Wild	0	0.000	.	.	.	0.98	0.95	1.02
Origin × R	Hatchery	1	0.140	0.019	57.1	<0.001	1.34	1.31	1.38
	Wild	0	0.000	.	.	.	1.17	1.13	1.21
Origin × SLP	Hatchery	1	0.051	0.010	28.8	<0.001	1.13	1.10	1.15
	Wild	0	0.000	.	.	.	1.07	1.05	1.09

### Adult oysters

**Figure 6:**
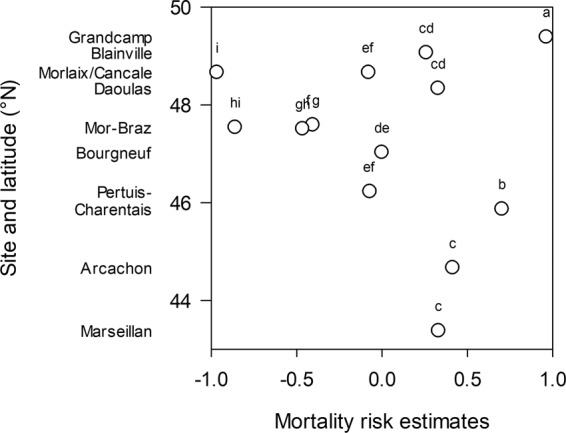
Mortality risk estimates of adult oysters as a function of latitude or sites. Letters indicate significant differences.

Adult mortality risk varied considerably among sites but was not correlated with latitude (Fig. 6, Tables 3 and S4). Detection of *Vibrio aestuarianus* DNA and seawater temperature were associated with a higher mortality risk in adults whereas salinity and precipitation were associated with a lower risk (Tables 3 and S4). The detection of *V. aestuarianus* was the most important factor associated with mortality risk in adults.

**Table 3:** Model parameter estimates from Cox regression models using latitude (univariate model) or time-dependent environmental parameters and pathogen detection selected by the stepwise procedure for adult oysters (multivariate model).

Parameter	Df	Estimate	SE	χ^2^	p	Odds ratio	95% CI	
Model 1: Latitude	1	−0.025	0.059	3.4	0.669	0.98	0.87	1.10
**Model 3: Environment and pathogen**								
*V. aestuarianus*	1	1.045	0.125	69.5	<0.001	2.85	2.23	3.64
Temperature	1	0.048	0.016	8.5	0.004	1.05	1.02	1.08
Salinity	1	−0.033	0.016	4.3	0.038	0.97	0.94	1.00
Rainfall	1	−0.063	0.020	10.1	0.002	0.94	0.90	0.98

## Discussion

In this study, we deciphered the complex interaction of host, pathogen and environmental factors on mortality of oysters along a latitudinal gradient. More particularly, we found that mortality risk in young oysters from the hatchery was associated with OsHV-1 and increased southward. This result is in line with the general idea that latitudinal gradient influences the likelihood of disease outbreaks^21,22^. This also agrees well with a study conducted before the emergence of the μVar genotype of OsHV-1 in France^31^.

Here we found that mortality risk in young oysters from the hatchery was associated with environmental parameters measured before and during the mortality events. Among them, temperature played a major role. We indeed confirmed that mortality associated with OsHV-1 started in oysters at almost all sites when seawater temperature reached ∼16 °C^8,9,32^. The lower the temperature before the onset of mortality and the higher the temperature after, the higher the risk of mortality. Therefore, the rate of temperature increases, up until reaching 16 °C and thereafter during the season, can be considered a risk factor of OsHV-1-induced mortality in oysters. Increased and more variable temperatures have been associated with disease outbreaks in numerous invertebrate species, including corals^33,34^, echinoderms^35^, and shellfish^36^. However, the mechanisms for temperature facilitation of disease progression in oysters are currently unknown.

We also found that food quantity measured before and during the mortality event was associated with a higher mortality risk of oysters. Although food availability generally improves the physiological condition of the host and lowers their susceptibility to infectious disease, reflecting a tradeoff between immunity and other functions^37,38^, food scarcity can limit the resources available to the pathogen and slow the growth and metabolism of the host on which the pathogen depends to proliferate^39–41^. In the context of oyster-OsHV-1 interaction, greater food availability favors the acquisition of energy reserves but overall increases mortality risk by increasing the growth and metabolism of the host^42^.

We discovered here that both rainfall and mean sea level atmospheric pressure measured before and during the mortality episode were associated with a higher mortality risk of oysters. This result seems somewhat paradoxical since these two variables were negatively correlated, indicating that in low atmospheric pressure areas, rising air cools, condenses, forms clouds and perhaps rain. However, although correlation was significant, the major part of the variance (58.1%) remained unexplained.

Although speculative, the relationship between rainfall and mortality risk may reflect the effect of increased terrestrial inputs from watershed and turbidity on disease susceptibility of oysters^15^. Indeed, suspended particulate matter may facilitate disease transmission by providing micro-environment for viruses and by attenuating the penetration of ultraviolet irradiance in the water column which reduces virus infectivity^43,44^. Also, terrestrial inputs are generally of poor nutritional quality and reduce feeding efficiency and energy intake of oysters^45^, which can in turn affect disease susceptibility.

According to our model, high atmospheric pressure, which tends to be associated cloudless skies, was associated with increasing mortality risk of young oysters. This may simply reflect the fact that OsHV-1 outbreaks occur typically in late spring and early summer when the weather becomes mild and temperature increases.

Finally, we found that wind speed measured before and during the mortality event was associated with a higher mortality risk of oysters. Wind, alongside with tides, bathymetry, freshwater inputs and sea level atmospheric pressure, is a major component of the hydrodynamic regime that influences the speed and direction of surface currents. Wind speed potentially increases the connectivity between farms and sites, and thus, the risk of transmission of pathogens as reported in sea lice infecting salmon^46,47^. Additionally, in coastal ecosystems, increasing wind speed favors the resuspension of sediments and the increase in turbidity, which is a risk factor for mortality^15^.

The mortality risk of wild oysters was not related to latitude, and sites and environmental factors exert a much lower influence than for hatchery oysters. Similarly, rearing height has a major influence on mortality risk of hatchery oysters but not much on wild animals^23^. Like in the present study, wild oysters were asymptomatic carriers of the virus whereas hatchery oysters were free of OsHV-1. Therefore, the lower influence of latitude, site, or environmental factors on the mortality risk in wild oysters probably reflect that a thermal elevation beyond the permissive threshold (16 °C in Europe) was sufficient to reactivate the virus in these asymptomatic carriers^12,13^. Additional stressors like rate of temperature increase, food availability, rainfall, sea level atmospheric pressure and wind speed were of lower importance.

Survival of hatchery oysters was higher than that of wild oysters at latitude >47.6°N but at lower latitudes the opposite was true. At latitude >47.6°N, environmental conditions may have partially protected hatchery oysters from pathogens, whereas wild oysters most likely died because the virus reactivated when seawater temperature reached 16 °C. Conversely, at lower latitude, hatchery oysters were at higher risk of mortality because the presumed protection from the environment was decreased, and their susceptibility to disease was greater than that of wild oysters. Indeed, wild oysters are more likely to have been exposed to the pathogen at the larval and post-larval stages, and thus selected for greater resistance to the disease. It is possible, however, that different genetic backgrounds were associated with oyster origins and contributed to the interactive effect of latitude and the origin of oyster.

Finally, we found that mortality risk in adults was associated with *V. aestuarianus* and varied considerably among sites but not consistently with latitude. It more likely reflects habitat characteristics like biotic (e.g. species composition and abundance) and abiotic parameters (depth, substratum, current, salinity, etc.) that are not necessarily correlated with latitude. The risk factors involved in young and adult oyster mortalities were partly different, reflecting distinct pathogens^14^.

Among them, increasing seawater temperature and decreasing salinity were associated with a higher mortality risk in adults, likely reflecting physiological imbalance and increased susceptibility to pathogens^48^. This agrees with the fact that mortality events recorded along the Atlantic coast of France usually occur months after winters dominated by the occurrence of positive North Atlantic oscillation (NAO+) atmospheric regimes of circulation^49^. The NAO+ is characterized by positive anomalies in air temperature and rainfall leading to higher sea surface temperature and higher river flow and lower salinity^48^.

We finally observed that increasing rainfall decreased mortality risk for adult oysters. This may appear paradoxical if we consider that rainfall enhance freshwater inputs and lower salinity. The relationship between rainfall and mortality risk in adults may reflect other side effects that require further investigation.

In conclusion, we found that latitudinal gradient alters disease-induced mortality risk in oysters but this effect interacts with the initial health status of the host and the pathogen involved. From a practical point of view, we suggest that young oyster mortality in the north can be mitigated by favoring seed from hatcheries that are more likely to be free of OsHV-1. Conversely, to limit mortality in the south, it seems preferable to raise wild spat that have often been exposed to the virus in their early life stages and are therefore less susceptible to the disease.

## Supplementary information

Supplementary Information.

Supplementary Information 2

## Data Availability

All data are available in supplementary information file and in data-complete.xls.
