# Supplementary material for: Latitudinal drivers of oyster mortality: deciphering host, pathogen and environmental risk factors
Source: Sci Rep. 2020 Apr 29;10:7264. doi: 10.1038/s41598-020-64086-1 (PMC7190702; doi:10.1038/s41598-020-64086-1)
Supplement: Supplementary file 1 — Supplementary Information. [file 41598_2020_64086_MOESM1_ESM.docx]

**Latitudinal drivers of oyster mortality: deciphering host, pathogen and environmental risk factors**

**Elodie Fleury^1*^, Pierrick Barbier^1^, Bruno Petton^1^, Julien Normand^2^, Yoann Thomas^3^, Stéphane Pouvreau^1^, Gaétan Daigle^4^, Fabrice Pernet^1^^[[1]](#footnote-1)^**

^1^ Ifremer, CNRS, IRD, Univ Brest, LEMAR, F-29280 Plouzane, France

^2^ Ifremer/ Laboratoire Environnement Ressources de Normandie, 14520 Port en Bessin, France

^3^ IRD, CNRS, Ifremer, Univ. Brest, LEMAR, F-29280 Plouzane France

^4^ Département de Mathématiques et Statistique, Université Laval, Sainte-Foy, Québec, G1K 7P4, Canada

* [elodie.fleury@ifremer.fr](mailto:elodie.fleury@ifremer.fr)

SUPPORTING INFORMATION

Table S1. Model parameter estimates from Cox regression models using site as fixed factor for young oysters.

| **Parameter** | **Level** |  | **Df** | **Estimate** | **SE** | **χ2** | **P** |
| --- | --- | --- | --- | --- | --- | --- | --- |
| Site† | Agnas |  | 1 | 0.174 | 0.134 | 1.7 | 0.192 |
|  | Blainville |  | 1 | 0.600 | 0.114 | 27.5 | <0.001 |
|  | Brest |  | 1 | 0.620 | 0.124 | 25.0 | <0.001 |
|  | Cancale |  | 1 | 0.286 | 0.126 | 5.2 | 0.023 |
|  | Coupelasse |  | 1 | 0.237 | 0.136 | 3.0 | 0.082 |
|  | Gefosse |  | 1 | -0.119 | 0.127 | 0.9 | 0.352 |
|  | LarmorBaden |  | 1 | 0.239 | 0.118 | 4.1 | 0.043 |
|  | Loix |  | 1 | 0.691 | 0.129 | 28.5 | <0.001 |
|  | Marseillan |  | 1 | 0.746 | 0.130 | 32.8 | <0.001 |
|  | MenErRoue |  | 1 | 0.318 | 0.108 | 8.7 | 0.003 |
|  | Morlaix |  | 1 | -0.013 | 0.122 | 0.0 | 0.913 |
|  | Penerf |  | 1 | 0.288 | 0.124 | 5.4 | 0.021 |
| Origin (O)‡ | Hatchery |  | 1 | 0.506 | 0.121 | 17.6 | <0.001 |
| Site x O | Agnas | Hatchery | 1 | -0.034 | 0.170 | 0.0 | 0.841 |
|  | Blainville | Hatchery | 1 | -1.043 | 0.153 | 46.6 | <0.001 |
|  | Brest | Hatchery | 1 | -0.712 | 0.164 | 18.7 | <0.001 |
|  | Cancale | Hatchery | 1 | -1.117 | 0.159 | 49.3 | <0.001 |
|  | Coupelasse | Hatchery | 1 | 0.302 | 0.169 | 3.2 | 0.073 |
|  | Gefosse | Hatchery | 1 | -0.313 | 0.159 | 3.9 | 0.049 |
|  | LarmorBaden | Hatchery | 1 | -0.476 | 0.150 | 10.1 | 0.002 |
|  | Loix | Hatchery | 1 | -0.084 | 0.158 | 0.3 | 0.594 |
|  | Marseillan | Hatchery | 1 | -0.214 | 0.169 | 1.6 | 0.205 |
|  | MenErRoue | Hatchery | 1 | -0.638 | 0.139 | 21.0 | <0.001 |
|  | Morlaix | Hatchery | 1 | -0.517 | 0.156 | 11.0 | 0.001 |
|  | Penerf | Hatchery | 1 | -0.640 | 0.160 | 16.1 | <0.001 |

† The reference site is Arcachon

‡ The reference origin is wild

Table S2. Model parameter estimates from univariate Cox regression models using environmental parameters as covariates for young oysters. The average of each environmental variable was calculated over the longest period preceding the mortalities (15d for wild and 50d for hatchery oysters).

| **Effect** | **Df** | **χ2** | **p** |
| --- | --- | --- | --- |
| Origin (O) | 1 | 58.6 | <0.001 |
| Temperature (T) | 1 | 146.5 | <0.001 |
| O x T | 1 | 61.6 | <0.001 |
|  |  |  |  |
| O x T | 1 | 27.3 | <0.001 |
| Food level (F) | 1 | 19.8 | <0.001 |
| O x F | 1 | 32.5 | <0.001 |
|  |  |  |  |
| O x T | 1 | 26.7 | <0.001 |
| Salinity (S) | 1 | 228.8 | <0.001 |
| O x S | 1 | 26.9 | <0.001 |
|  |  |  |  |
| O | 1 | 17.8 | <0.001 |
| Sea-level pressure (SLP) | 1 | 156.0 | <0.001 |
| O x SLP | 1 | 17.8 | <0.001 |
|  |  |  |  |
| O | 1 | 19.7 | <0.001 |
| Rainfall | 1 | 185.0 | <0.001 |
| O x R | 1 | 22.7 | <0.001 |
|  |  |  |  |
| O | 1 | 7.6 | 0.006 |
| Wind speed (W_WE_) | 1 | 109.6 | <0.001 |
| O x W_WE_ | 1 | 49.4 | <0.001 |
|  |  |  |  |
| O | 1 | 3.3 | 0.068 |
| Wind speed (W_NS_) | 1 | 95.4 | <0.001 |
| O x W_NS_ | 1 | 1.7 | 0.190 |

Table S3. Summary of univariate Cox regression models using time-dependent environmental parameters and pathogen detection for young oysters.

| **Effect** | **Df** | **χ2** | **p** |
| --- | --- | --- | --- |
| Origin (O) | 1 | 223.6 | <0.001 |
| OsHV-1 | 1 | 1378.1 | <0.001 |
| O x OsHV-1 | 1 | 47.4 | <0.001 |
|  |  |  |  |
| O | 1 | 35.3 | <0.001 |
| *V. aestuarianus* (Va) | 1 | 0.4 | 0.548 |
| O x Va | 1 | 8.4 | 0.004 |
|  |  |  |  |
| Origin (O) | 1 | 411.4 | <0.001 |
| Temperature (T) | 1 | 117.5 | <0.001 |
| O x T | 1 | 358.7 | <0.001 |
|  |  |  |  |
| O | 1 | 48.1 | <0.001 |
| Food level (F) | 1 | 671.5 | <0.001 |
| O x F | 1 | 21.0 | <0.001 |
|  |  |  |  |
| O | 1 | 96.7 | <0.001 |
| Salinity (S) | 1 | 1.0 | 0.314 |
| O x S | 1 | 91.6 | <0.001 |
|  |  |  |  |
| O | 1 | 34.9 | <0.001 |
| Wind speed (W_WE_) | 1 | 10.6 | 0.001 |
| O x W_WE_ | 1 | 6.2 | 0.013 |
|  |  |  |  |
| O | 1 | 24.3 | <0.001 |
| Wind speed (W_NS_) | 1 | 3.6 | 0.058 |
| O x W_NS_ | 1 | 6.5 | 0.011 |
|  |  |  |  |
| O | 1 | 47.0 | <0.001 |
| Sea-level pressure (SLP) | 1 | 1.4 | 0.233 |
| O x SLP | 1 | 46.7 | <0.001 |
|  |  |  |  |
| O | 1 | 0.2 | 0.625 |
| Rainfall | 1 | 266.2 | <0.001 |
| O x R | 1 | 10.5 | 0.001 |

Table S4. Model parameter estimates from Cox regression models using site as fixed factor for adult oysters.

| **Parameter** | **Level** |  | **Df** | **Estimate** | **SE** | **χ2** | **P** | **Odds ratio** |
| --- | --- | --- | --- | --- | --- | --- | --- | --- |
| Site | Agnas |  | 1 | 0.288 | 0.124 | 5.388 | 0.020 | 1.334 |
|  | Blainville |  | 1 | -0.156 | 0.140 | 1.246 | 0.264 | 0.856 |
|  | Brest |  | 1 | -0.086 | 0.137 | 0.394 | 0.530 | 0.918 |
|  | Cancale |  | 1 | -1.381 | 0.202 | 46.771 | <0.001 | 0.251 |
|  | Coupelasse |  | 1 | -0.415 | 0.152 | 7.421 | 0.006 | 0.660 |
|  | Gefosse |  | 1 | 0.548 | 0.118 | 21.460 | <0.001 | 1.730 |
|  | LarmorBaden |  | 1 | -0.820 | 0.169 | 23.419 | <0.001 | 0.440 |
|  | Loix |  | 1 | -0.486 | 0.154 | 9.999 | 0.002 | 0.615 |
|  | Marseillan |  | 1 | -0.083 | 0.140 | 0.356 | 0.551 | 0.920 |
|  | MenErRoue |  | 1 | -1.275 | 0.196 | 42.452 | <0.001 | 0.279 |
|  | Morlaix |  | 1 | -0.493 | 0.150 | 10.793 | 0.001 | 0.611 |
|  | Penerf |  | 1 | -0.880 | 0.174 | 25.682 | <0.001 | 0.415 |
|  | Tes |  | 0 | 0.000 | . | . | . | . |

Table S5. Summary of univariate Cox regression models using time-dependent environmental parameters and pathogen detection for adult oysters.

| **Effect** | **Df** | **χ2** | **p** |
| --- | --- | --- | --- |
| OsHV-1 | 1 | 0.2 | 0.620 |
| *V. aestuarianus* | 1 | 88.1 | <0.001 |
| Temperature | 1 | 8.8 | 0.003 |
| Salinity | 1 | 14.0 | 0.000 |
| Food level | 1 | 0.3 | 0.593 |
| Rainfall | 1 | 11.2 | 0.001 |
| Sea-level pressure | 1 | 0.6 | 0.440 |
| Wind speed (W_WE_) | 1 | 2.8 | 0.094 |
| Wind speed (W_NS_) | 1 | 0.1 | 0.756 |


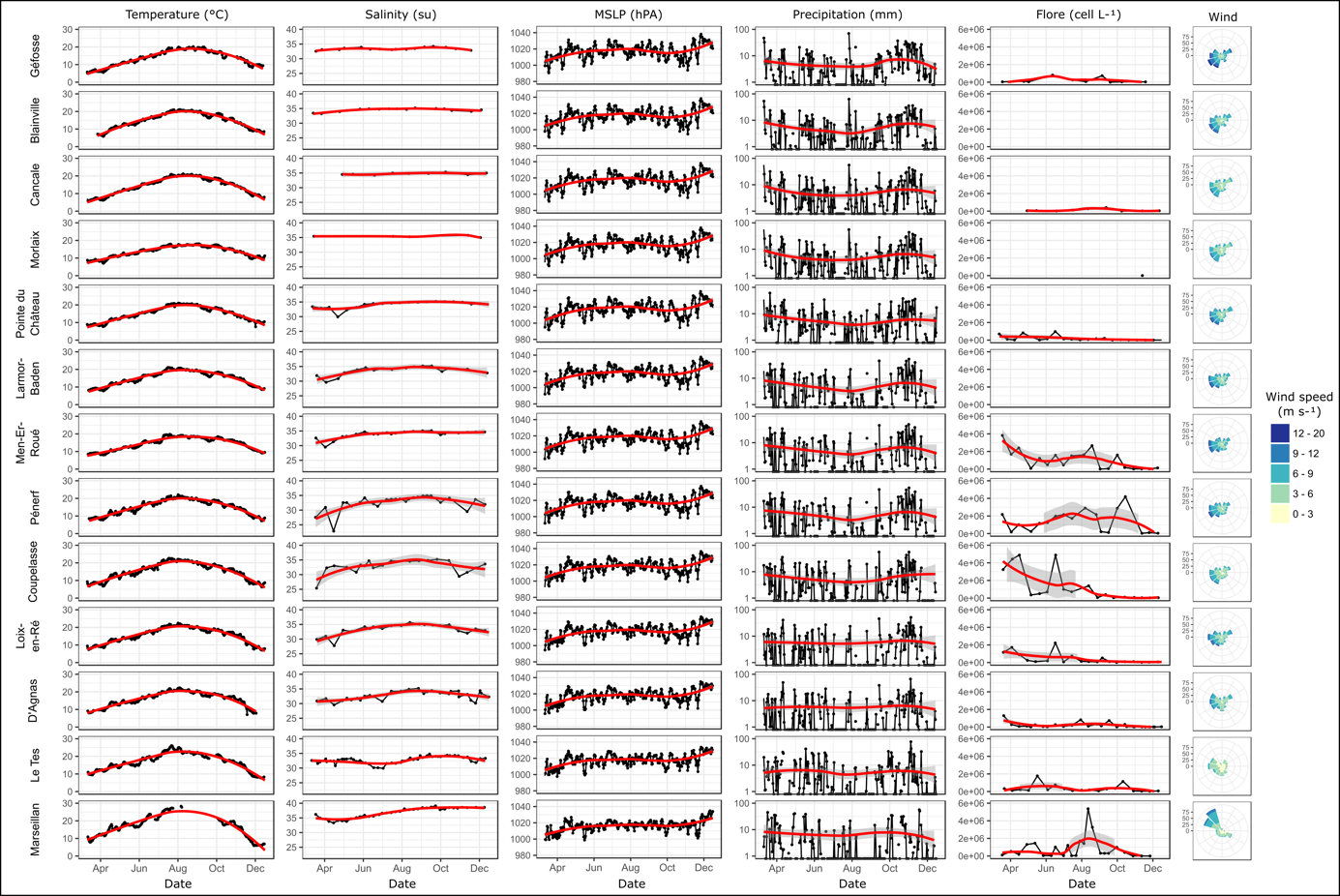


Figure S1. Environmental data.

1. [↑](#footnote-ref-1)
